# Predicting Vaccination Intention against COVID-19 Using Theory of Planned Behavior: A Systematic Review and Meta-Analysis

**DOI:** 10.3390/vaccines10122026

**Published:** 2022-11-26

**Authors:** Yam B. Limbu, Rajesh K. Gautam, Wencang Zhou

**Affiliations:** 1 Feliciano School of Business, Montclair State University, 1 Normal Ave., Montclair, NJ 07043, USA; 2 Department of Anthropology, Dr. Harisingh Gour Central University, University Road, Sagar 470003, MP, India

**Keywords:** vaccination intention, COVID-19, theory of planned behavior, attitude, subjective norms, perceived behavioral control, systematic review, meta-analysis

## Abstract

This study systematically analyzed the literature using the theory of planned behavior (TPB) as a theoretical framework to examine the influence of its constructs on vaccination intention against COVID-19. Quantitative studies were searched in PubMed, CINAHL, Web of Science, and Google Scholar following the PRISMA guidelines. The average rate of COVID-19 vaccination intention was 73.19%, ranging from 31% to 88.86%. Attitude had the strongest association with vaccination intention (*r_+_* = 0.487, 95% CI: 0.368–0.590), followed by subjective norms (*r_+_* = 0.409, 95% CI: 0.300–0.507), and perceived behavioral control (*r_+_* = 0.286, 95% CI: 0.198–0.369). Subgroup analyses showed that the pooled effect sizes of TPB constructs on vaccination intention varied across geographic regions and study populations. Attitude had large effect sizes in Asia, Europe, and Oceania, especially among the adult general population, parents, and patients. Subjective norms had large effect sizes in Asia and Oceania, especially among parents and patients. Perceived behavioral control was the most dominant predictor of vaccination acceptance in Africa among patients. These findings suggest that TPB provides a useful framework for predicting intention to receive a COVID-19 vaccine. Hence, public awareness and educational programs aimed at promoting COVID-19 vaccination intention should consider using TPB as a framework to achieve the goal.

## 1. Introduction

The recent COVID-19 pandemic has posed global challenges and a threat to humanity. Hence, in March 2020, the World Health Organization (WHO) declared it a pandemic [1]. Nevertheless, the impact of the pandemic was very distressing; as of 19 September 2022, there were over 609 million confirmed cases of COVID-19 globally, with over 6 million deaths. However, over 12 billion vaccine doses have been administered [2]. Usually, vaccine development takes an average of 10 years; however, in the case of COVID-19, several vaccine candidates entered into clinical trials within 6 months and were conditionally approved in 10 months [3]. More than 287 potential vaccines are being developed, and over 102 clinical trials were recently released [4,5]. According to the WHO, on 22 October 2021, there were 322 vaccine candidates in development. Around 40% were in clinical development (128 vaccine candidates), while 194 were in preclinical development [6]. Despite this success in the development of vaccines, almost one billion people in lower-income countries remain unvaccinated; only 57 countries have vaccinated 70% of their population, and almost all of them are high-income countries [7]. The WHO has a target to reach 70% vaccination coverage as soon as possible, including 100% for those aged over 60 years, health workers, and those with underlying conditions [7]; however, convincing individuals to accept vaccination against COVID-19 remains a major challenge. Similarly, improving vaccination rates, especially booster vaccination among specific groups such as children, is an immense obstacle in some countries such as China [8].

Vaccine hesitancy, which refers to a delay in the acceptance or a refusal of safe vaccines despite the availability of vaccination services [9], has been the major barrier to COVID-19 vaccine acceptance. Previous studies showed that many people were hesitant to get vaccinated across the world, such as 37.3% in Uganda [10], 64% in Egypt [11], and 23% in the United States [12]. Limbu et al. [13] also reported an overall vaccination hesitancy rate for COVID-19 of 33.23%, with the highest rate in France (60.6%), followed by China (56.4%), South Korea (53.3%), Bangladesh (46.2%), and the United States (43.5%). They also found that vaccine hesitancy was more prevalent among diabetes patients (56.4%), while the lowest vaccine hesitancy was reported among healthcare workers (15%). Vaccine hesitancy is considered one of the greatest threats to the ongoing COVID-19 vaccination programs and to the progress in tackling the disease [9,14]. In order to achieve a higher coverage of the vaccines, it is essential to elicit a positive attitude toward the vaccine amongst individuals and populations [15]. Therefore, it is imperative to identify the causes of refusal/hesitancy and accordingly make suitable interventions [15]; on the other hand, it is essential to identify the factors helping in fostering positive intentions toward the uptake of vaccines. Since vaccination intention, which refers to the intention to take a vaccine when offered [16], is pivotal to the success of vaccination campaigns to attain herd immunity, it is essential to understand the factors influencing COVID-19 vaccination intention.

Prior studies showed that COVID-19 vaccination intention ranged from 67% to 91% across countries such as India, Saudi Arabia, Canada, the United States, and China [17,18,19,20,21,22]. Various factors are related to willingness to accept the COVID-19 vaccine, including socioeconomic factors [23,24], psychological determinants [25,26], and informational aspects such as the role of availability of information and misinformation on vaccination intention [23,24,25,27]. Several demographic factors and perception of the disease’s risk have been found to be associated with COVID-19 vaccination intention [21,22,28]. People’s perceptions of health risk, that they are more susceptible to infection of the disease, that it is a serious threat to their health, and that the vaccine will successfully protect them are more likely to get vaccinated [29]. Similarly, a health provider’s recommendation, which is a type of subjective norm, may also impact vaccine uptake [30].

Several theories have been used to predict COVID-19 vaccination intention, including the theory of planned behavior (TPB) [31]. The TPB, proposed by Icek Ajzen as a successor of the theory of reasoned action [32], is one of the best-supported social psychological theories in relation to predicting human behavior in different populations and contexts [33,34,35]. The TPB holds that behavioral intentions are the outcome of a combination of three factors: attitudes about the behavior, subjective norms (i.e., social influence/pressure on people to perform or not to perform the particular behavior), and perceived behavioral control (i.e., an individual’s perception of their ability to perform the behavior). It has been proposed as a theoretical guideline to explain the factors influencing various health behaviors in public health research [36,37]. The basis of the TPB is that we make systematic use of available information and consider the consequences of our actions before engaging in a behavior [38]. With a strong intention to carry out a behavior, a person tends to perform that behavior [38]. According to Ajzen [37], the complexities of the health behaviors can be successfully dealt with by TPB. Hence, the objective of this systematic review and meta-analysis was to analyze the literature using the TPB as a theoretical framework to investigate the role of its constructs in determining the intention to get vaccinated against COVID-19.

## 2. Previous Systematic Reviews and Meta-Analyses

Several systematic reviews have already been conducted on vaccination intention against COVID-19. These reviews analyzed COVID-19 vaccination intentions across genders [39] and healthcare workers [40], as well as between healthcare workers and the general population [41]. Two studies conducted rapid reviews, a simplified approach to systematic reviews [42,43]. Some studies performed scoping reviews to explore broad factors such as demographic, social, and contextual factors that influenced the intention to use COVID-19 vaccines [44]. Patwary et al. [45] performed a rapid systematic review and meta-analysis to summarize the COVID-19 vaccine acceptance rates and factors associated with acceptance in low- and lower-middle-income countries. In a scoping review, Willems et al. [46] provided some insight into the factors influencing COVID-19 vaccine hesitancy and the willingness of healthcare workers, including those who care for people with intellectual disabilities. Wang et al. [47] and Chen et al. [48] estimated the COVID-19 vaccine acceptance rate and identified predictors associated with COVID-19 vaccine acceptance. Shakeel et al. [49] conducted a systematic review to examine how and why the rates of COVID-19 vaccine acceptance and hesitancy differ across countries and continents. Sallam et al. [50] conducted a concise narrative review and provided an updated perspective on the status of COVID-19 vaccine acceptance rates worldwide. Roy et al. [51] conducted a systematic review to identify factors influencing COVID-19 vaccine acceptance and refusal intention. Renzi et al. [52] conducted a meta-analysis to explore the prevalence of COVID-19 vaccine acceptance with a specific focus on worldwide geographical differences. Terry et al. [53] conducted a systematic review and meta-analysis of cross-sectional studies to identify factors associated with public intention to receive COVID-19 vaccines until February 2021. Alarcón-Braga et al. [54] performed a systematic review to estimate the prevalence of the intention to vaccinate against COVID-19 in Latin America and the Caribbean and to explore how it varies across different age groups. In conclusion, prior systematic reviews mainly focused on narrow topics and rapid, scoping, or narrative reviews. However, to the best of our knowledge, no systematic review and meta-analysis has reviewed the literature using TPB as a theoretical framework and addressed TPB’s utility in predicting vaccination intention against COVID-19.

The current study contributes to the literature in several ways. Firstly, to our knowledge, this study represents the first systematic review and meta-analysis of quantitative studies examining the association between TPB constructs and COVID-19 vaccination intention. Secondly, this review and meta-analysis identifies the occurrence of the TPB constructs that are positively associated with behavioral intention to vaccinate against COVID-19. Furthermore, these results are broken down by year of study, geographical region, and population type. The subgroup meta-analyses are performed to examine the impacts of TPB constructs on vaccination intention across geographic regions and study populations. Thirdly, this study provides an up-to-date and comprehensive review of the latest studies, including articles published in 2022, and those articles covering booster/third-dose vaccination intention. Fourthly, this review and meta-analysis also includes the studies that examined parents’ or caregivers’ intention to vaccinate their young children against COVID-19. Lastly, we report on the overall vaccination intention rate by types of vaccines (original shots vs. boosters), country and continent, year, and population type.

## 3. Methodology

For this systematic review and meta-analysis, we followed the guidelines of the Preferred Reporting Items for Systematic Reviews and Meta-Analyses (PRISMA) [55,56]. We searched four databases for articles using the theory of planned behavior to examine COVID-19 vaccination intentions using key terms such as “theory of planned behavior” or “TPB”, “COVID-19”, “corona virus”, “booster shot or dose”, “SARS-CoV-2”, and “vaccination intention”. The search was conducted from 3 January 2022 to 15 August 2022. Full-length papers published during December 2019 and August 2022 were retrieved for analysis.

### 3.1. Inclusion and Exclusion Criteria

The main inclusion criteria were quantitative studies published in peer-reviewed journals, written in English, that used the TPB as a theoretical basis to examine the associations between TPB constructs and COVID-19 vaccination intention. We excluded qualitative studies, non-peer-reviewed studies, conference proceedings, and case reports.

### 3.2. Search Strategy

We conducted a comprehensive search of the published literature using each of the four selected databases: PubMed (National Library of Medicine), Web of Science (Clarivate), CINAHL, and Google Scholar. The combinations of key terms and Boolean operators (AND, OR) that were used to locate studies in each database are presented in Table 1.

Two researchers independently screened the titles and abstracts of the identified articles. Non-quantitative studies, and the studies not applying the TPB framework for predicting vaccination intention, were excluded. Full-text articles were obtained for studies whose titles and abstracts met the inclusion criteria. Then these full-text articles were evaluated to confirm if they reported the necessary statistics of TPB constructs with respect to vaccination intention.

**Figure 1:**
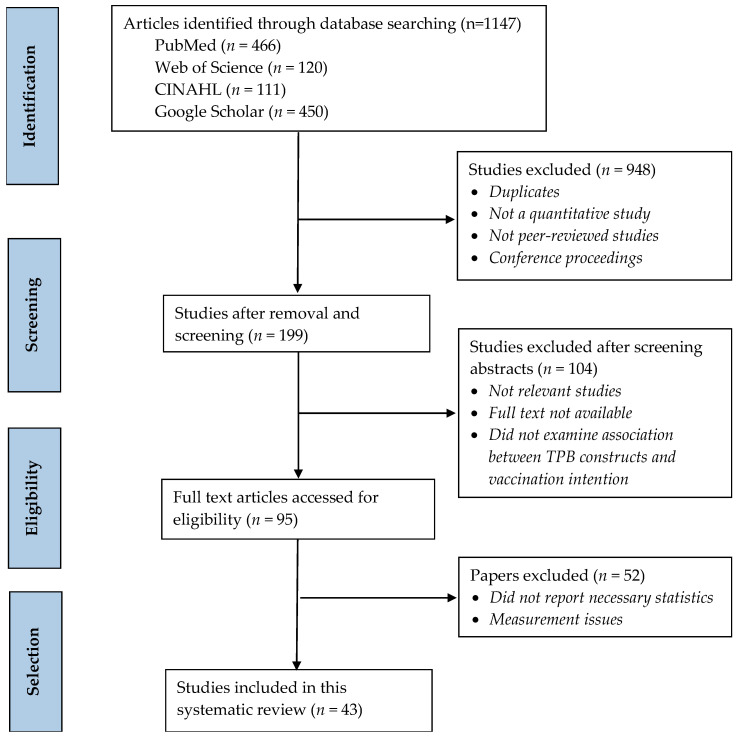
PRISMA flow diagram showing search strategy and study selection process.

PRISMA flow diagram demonstrates the study selection process, the number of records identified, screened, and excluded, and the reasons for exclusion (see Figure 1). A total of 1147 records were retrieved from the electronic databases. Of them, 948 records were removed as duplicates, conference proceedings, qualitative studies, and non-peer-reviewed articles. A total of 104 articles were excluded after screening the abstracts for being irrelevant or not examining the relationships between TPB constructs and vaccination intention. The remaining 95 full-text articles were further assessed for eligibility, and only 43 studies were found eligible for this systematic review and meta-analysis.

### 3.3. Data Extraction and Analysis

The same two researchers extracted data independently of one another. The following information was extracted from each study: author’s name, data collection year, publication year, study objective, study design, participants, sample size, sampling method, measures, statistical analysis techniques, analytical tools, country where the study was conducted, statistics (e.g., effect size, odd ratio, means, and standard deviations), and vaccination rate. We also extracted information on TPB constructs associated with vaccination intention. 

Data were analyzed using IBM SPSS Statistics 27 and Comprehensive Meta-Analysis 4.0. First, characteristics of studies included in the review were summarized using frequencies and percentages. Average vaccination intention rates were reported by country, sample, and year of data collection. The prevalence and strengths of TPB constructs that were significantly related to vaccination intention were presented by year, geographical region, and population. The effect size metric reported in this meta-analysis was the sample-weighted average correlation (*r*_+_). The included studies used different types of effect sizes, such as correlation coefficients, multiple regression coefficients, and odds ratios. Odds ratios were converted into correlation coefficients [57]. When a study neither reported the odds ratios nor the correlation coefficients, the reported standardized regression coefficients were used to calculate the effect size [58]. A random effect model was used for the meta-analyses as the samples in the included studies were heterogeneous [59]. The within-study variation was estimated with a 95% confidence interval (CI) and the between-study variation was estimated with the maximum likelihood estimator for tau (the standard deviation of true effect sizes). The Higgins and Thompsons’ [60] I^2^ was used to assess heterogeneity. Subgroup analyses on the geographic location (continent) of the study and sample population were conducted to explore the sources of heterogeneity.

## 4. Results

### 4.1. Study Characteristics

Forty-three studies were included in this systematic review and meta-analysis. Twenty-six of them were published in 2021, sixteen were published in 2022, and one was published in 2020 (see Table 2). However, most studies (24/43) collected data in 2021, sixteen collected data in 2020, and one collected data in 2022. Nearly half of the studies (21/43) were conducted in Asia, in contrast to nine in North America, nine in Europe, three in Africa, and one in Oceania. However, astonishingly, no study was carried out in South America. This review and meta-analysis included studies from twenty countries, including nine from the United States, nine from China, and three from India. Forty-one studies were cross-sectional in design. The studies included in this review and meta-analysis consisted of 64,359 respondents, with an average sample size of 1496 respondents (standard deviation = 2380.71), ranging from 69 [61] to 11,141 [62]. The majority of the studies focused on the general adult population (69.8%), followed by patients (9.2%), students (7%), healthcare workers (4.6%), parents (4.7%), and factory workers (4.7%).

### 4.2. Vaccination Intention Rate

The average rate of COVID-19 vaccination intention was 73.19% (SD = 11.63), ranging from 31% [62] to 88.86% [31]. However, vaccination acceptance for a booster shot was much higher (85.51%, SD = 2.54) than that for the original shot(s) (72.48%, SD = 11.56). The vaccination intention appeared slightly higher in Western countries (74.49%) than in non-Western countries (72.2%). There was no significant difference between US and Chinese adults in vaccination intention (74.28% versus 75.81%). Vaccination acceptance rate slightly increased in 2021 (75.89%) from 2020 (72.61%). Vaccine acceptance was higher among patients (80.4%), followed by students (78.37%), healthcare workers (75.33%), and the general adult population (72.74%). Vaccine acceptance was lower among parents for their children (59.15%).

### 4.3. TPB Constructs Associated with Vaccine Intention

Table 2 presents the frequency of significant relationships between TPB constructs and COVID-19 vaccination intention. Although all studies included in this systematic review and meta-analysis used the TPB as the theoretical basis, eight studies focused on only one or two of its constructs. Thirty-five studies (81.4%) used the TPB in its entirety. Our results show that people’s attitude toward COVID-19 vaccination was the most frequently demonstrated TPB construct influencing vaccination intention in thirty-eight studies (92.68%); however, three studies [63,64,65] showed an insignificant effect. Two studies did not include it as a predictor. Subjective norms were significantly associated with vaccination intention in thirty studies (78.57%), but the association was not statistically significant in nine studies (21.43%). In one study [66], it was not examined as a determinant. Perceived behavioral control was found to be directly associated with vaccination intention in twenty studies (55.56%); however, surprisingly, the association was not statistically significant in sixteen studies (44.44%). Seven studies did not include it as a model construct. Eight studies tested an extended TPB by incorporating self-efficacy, which was found to be a significant predictor of COVID-19 vaccination intention.

A few studies examined the role of moderators and mediators in the relationships between TPB constructs and vaccination intention. For example, Dou et al. [63] found that the association between attitude and vaccination intention was significantly stronger for Chinese males, whereas the association between subjective norms and vaccination intention was significantly stronger for Chinese females. However, gender difference was not evident in the relationship between perceived behavioral control and vaccination intention. One study [67] surveyed Italians and concluded that the effect of subjective norms on vaccination intention is fully mediated by trust in science. Ekinci et al. [68] showed that the effect of subjective norms on COVID-19 vaccination intention was partially mediated by attitude toward COVID-19 vaccines.

**Table 2:** Study Characteristics and TPB Constructs Influencing COVID-19 Vaccination Intention.

Author(s)	Year of Publication	Country	Vaccine Intention %	Population	Sample Size	Survey Year	TPB Construct—Vaccination Intention Association		
							ATT	SN	PBC
Almoayad et al. [69]	2022	Saudi Arabia	47.43	adult general population	487	2021	YES	YES	NS
An et al. [70]	2021	Vietnam	77.10	student	854	2021	YES	NS	YES
An et al. [71]	2021	Vietnam	80.50	patient	462	2021	YES	YES	YES
Asmare et al. [72]	2021	Ethiopia	64.90	adult general population	1080	2021	YES	YES	YES
Barattucci et al. [67]	2022	Italy	83.71	adult general population	1095	2021	YES	YES	RNR
Berg and Lin [73]	2021	USA	70.60	adult general population	350	2020	YES	YES	NS
Breslin et al. [74]	2021	Ireland	66.70	adult general population	439	2021	YES	NS	YES
Callow and Callow [31]	2021	USA	88.86	adult general population	583	2020	YES	YES	NS
Chu and Liu [75]	2021	USA	82.10	adult general population	934	2020	YES	YES	NS
Dou et al. [63]	2022	China	73.00	adult general population	405	2021	NS	YES	YES
Drążkowski and Trepanowski [76]	2021	Poland	61.14	adult general population	551	2020	YES	YES	YES
Ekinci et al. [68]	2022	USA	69.90	adult general population	1008	-	YES	YES	RNR
Fan et al. [77]	2021	China	75.86	Student	3145	2021	YES	NS	NS
Goffe et al. [78]	2021	England	62.20	adult general population	1660	2020	YES	YES	NS
Guidry et al. [79]	2021	USA	59.90	adult general population	788	2020	YES	YES	NS
Hagger and Hamilton [80]	2022	USA	-	adult general population	479	2021	YES	YES	YES
Hayashi et al. [81]	2022	USA	-	adult general population	172	2021	YES	NS	YES
Husain et al. [82]	2021	India	71.50	adult general population	400	2021	YES	YES	YES
Irfan et al. [66]	2021	Pakistan	-	adult general population	754	2020	YES	RNR	RNR
Kaida et al. [61]	2022	Canada	79.70	patient	69	2021	YES	YES	RNR
Khayyam et al. [83]	2022	Pakistan	-	healthcare worker	680	2021	YES	YES	YES
Li et al. [62]	2022	Hong Kong	31.00	parent	11141	2022	YES	YES	NS
Mir et al. [84]	2021	India	-	adult general population	254	-	YES	YES	RNR
Ogilvie et al. [85]	2021	Canada	79.80	adult general population	4948	2020	YES	YES	NS
Okai and Abekah-Nkrumah [86]	2022	Ghana	62.70	adult general population	362	2021	YES	NS	RNR
Patwary et al. [64]	2021	Bangladesh	85.00	adult general population	639	2021	NS	NS	NS
Prakash et al. [87]	2022	India	83.54	adult general population	228	2021	YES	YES	NS
Qi et al. [88]	2021	China	80.00	patient	350	2021	RNR	YES	NS
Rosental and Shmueli [89]	2021	Israel	82.15	student	628	2020	YES	NS	NS
Rountree and Prentice [90]	2021	Ireland	70.04	adult general population	1995	2020	YES	YES	RNR
Seddig et al. [91]	2022	Germany	-	adult general population	5044	2021	YES	YES	NS
Servidio et al. [92]	2022	Italy	81.40	patient	276	2021	YES	YES	YES
Shmueli [65]	2021	Israel	80.00	adult general population	398	2020	NS	YES	NS
Sieverding et al. [93]	2022	Germany	76.70	adult general population	1428	2020	YES	YES	YES
Thaker and Ganchoudhuri [94]	2021	New Zealand	82.40	adult general population	650	2021	YES	NS	NS
Twum et al. [95]	2021	Ghana	83.00	adult general population	478	2021	YES	YES	YES
Ullah et al. [96]	2021	Pakistan	59.80	adult general population	1034	2020	YES	YES	YES
Wolff [97]	2021	Norway	76.71	adult general population	1003	2020	YES	YES	YES
Yahaghi et al. [98]	2021	Iran	76.80	adult general population	10843	2021	YES	YES	YES
Zhang et al. [99]	2021	China	66.60	factory worker	2053	2020	YES	YES	YES
Zhang et al. [100]	2020	China	72.60	factory worker	2053	2020	YES	YES	YES
Zhong et al. [101]	2022	China	75.33	nurse	547	2021	YES	YES	YES
Zhou et al. [8]	2022	China	87.30	parent	1602	2021	YES	NS	YES

ATT: attitude; SN: subjective norms; PBC: perceived behavioral control; YES: significant; NS: not significant; RNR: result not reported.

**Figure 2:**
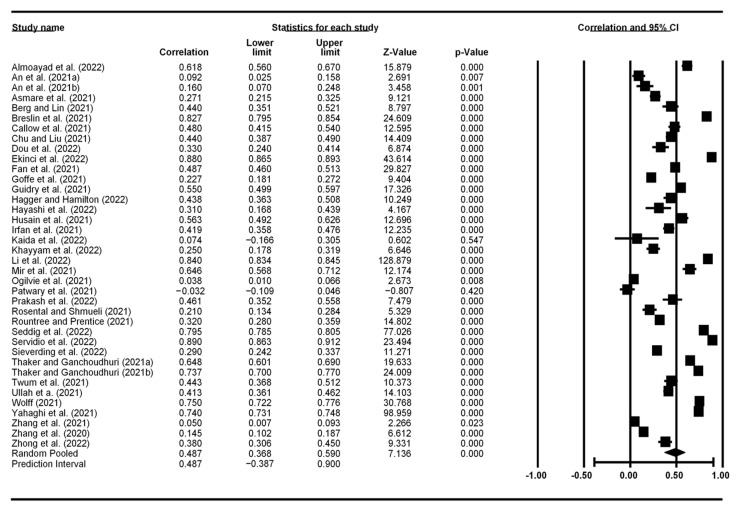
Forest plot showing attitude and vaccination intention correlation.

**Figure 3:**
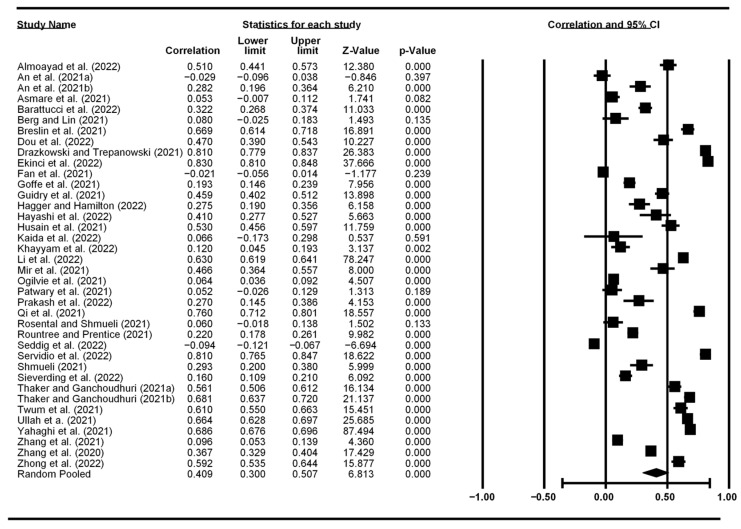
Forest plot showing subjective norms and vaccination intention correlation.

**Figure 4:**
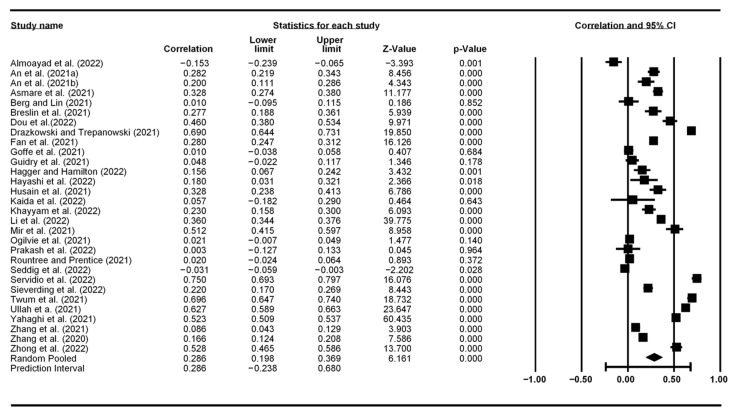
Forest plot showing perceived behavioral control and vaccination intention correlation.

**Figure 5:**
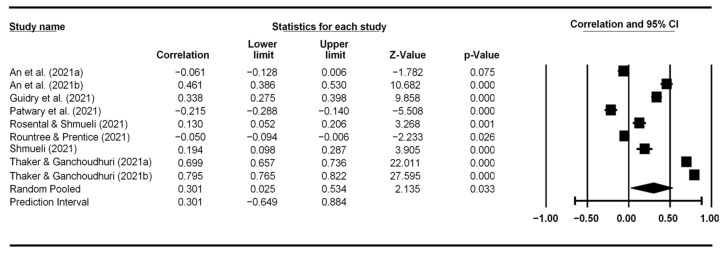
Forest plot showing self-efficacy and vaccination intention correlation.

Figure 2, Figure 3, Figure 4 and Figure 5 display the meta-analytic results and forest plots on the correlations between TPB constructs (including self-efficacy) and vaccination intention, respectively. Attitude had the strongest association with vaccination intention, yielding an average correlation of 0.487 (95% CI: 0.368–0.590). Subject norms had an effect size of 0.409 (95% CI: 0.300–0.507) on vaccination intention. Perceived behavioral control had the smallest effect size of 0.286 (95% CI: 0.198–0.369). Self-efficacy had an overall effect size of 0.301 (95% CI: 0.025–0.534).

We also performed subgroup analyses to explore the sources of high heterogeneity in the main analyses, in which I^2^ exceeded 75% for each effect size, indicating that the subgroups were very heterogeneous [102]. The breakdown of average effect sizes by moderators (geographic region and study population) are presented in Table 3, Table 4 and Table 5. The pooled effect sizes of TPB constructs on vaccination intention varied across geographic regions and study populations. 

In terms of geographic representation, attitude toward COVID-19 vaccine was a statistically significant predictor of vaccine acceptance in all eighteen studies in a Western context, but only 87% of the studies reported attitude as a significant determinant in a non-Western context. Furthermore, 85% of the Asian studies found a significant positive relationship between attitude and vaccine acceptance, but the association was significant in all studies conducted in North America, Europe, Africa, and Oceania. In sixteen out of nineteen studies (84.2%), subjective norms significantly predicted vaccination intention in Western countries, whereas only 73.9% of the studies (17/23) predicted vaccination intention in non-Western countries. Only 46.7% (7/15) of the studies in a Western setting and 61.9% (13/21) in a non-Western setting found a significant influence of perceived behavioral control on vaccination intention. Perceived behavioral control was found to be a key factor influencing vaccination intention in Africa. Subgroup analyses also revealed that effects of TPB constructs on vaccination intention differed by geographic region. Attitude had large and significant effect sizes on vaccination intention in Asia 0.65 [95% CI: 0.64, 0.66], Europe 0.63 [95% CI: 0.62, 0.63], and Oceania 0.70 [95% CI: 0.67, 0.72]. Subjective norms had large and significant effect sizes on vaccination intention in Asia 0.52 [95% CI: 0.51, 0.53] and Oceania 0.62 [95% CI: 0.59, 0.66]. While perceived behavioral control did not have a large impact in other subgroups, it was a significant predictor of vaccination intention in Africa, with an effect size of 0.46 [95% CI: 0.42, 0.50].

With regard to the study population, attitude predicted intention in all studies, except three studies that surveyed the general adult population [63,64,65]. Subjective norms were a more frequently demonstrated predictor of vaccination intention among patients (100%), healthcare workers (100%), and factory workers (100%) compared with the general adult population (82.8%). Interestingly, all three studies that surveyed students revealed an insignificant effect [70,77,89]. All studies that surveyed healthcare workers [83,101] and factory workers [99] showed a significant effect of perceived behavioral control on vaccination intention. Subgroup analyses also showed that the pooled effect sizes of TPB constructs on vaccination intention varied across different sample populations (see Table 3, Table 4 and Table 5). Attitude had large and significant effect sizes on vaccination intention in the adult general population 0.41 [95% CI: 0.40, 0.42], parents 0.84 [95% CI: 0.83, 0.85], and patients 0.53 [95% CI: 0.47, 0.57]. Subjective norms had large and significant effect sizes on vaccination intention in parents 0.63 [95% CI: 0.62, 0.64] and patients 0.59 [95% CI: 0.56, 0.63]. While perceived behavioral control did not have a large impact in other subgroups, it was a significant predictor of vaccination intention in the patient subgroup, with an effect size of 0.43 [95% CI: 0.37, 0.48].

In terms of the study year, attitude was a significant predictor of vaccination intention more frequently in 2021 (93.8%) than in 2020 (90.9%). On the contrary, subjective norms were a dominant predictor of vaccine acceptance in fourteen studies (93.3%) in 2020, but only 66.7% of the studies reported the same in 2021. Similarly, perceived behavioral control was found to influence vaccination intention more frequently in 2021 (66.7%) than in 2020 (42.9%). We also performed meta-regressions for data collection date and vaccination intention; however, none of the results were significant. 

Only two studies examined the effects of TPB constructs on vaccination intention for booster shots. All core TPB constructs were statistically significant, positive predictors of booster vaccine intentions among Americans [81]. Zhou et al. [8] examined the predictors of parents’ intentions regarding the COVID-19 booster vaccination for their children. Attitude and perceived behavioral control were positively associated with parents’ intentions.

Two studies focused on parents’ intention to vaccinate their children. Parent’s intention was stronger if they had higher levels of positive attitudes toward vaccinating their children and if they reported stronger subjective norms [62]. However, perceived behavioral control was not a significant predictor of vaccination intention. Zhang et al. [99] found that positive attitudes toward COVID-19 vaccination, perceived subjective norm (i.e., the perception that a family member would support them in having their children take up COVID-19 vaccination), and perceived behavioral control to have the children take up COVID-19 vaccination were associated with higher parental acceptability of COVID-19 vaccination. 

## 5. Discussion

Vaccination is recognized as the most successful and cost-effective public health intervention to combat the ongoing COVID-19 pandemic. Furthermore, it has made a significant contribution to improving global health by reducing the incidence and deaths of many infectious diseases [103,104]. Incidentally, despite the availability of vaccines and mass global drive for vaccination, many people remain hesitant to be vaccinated, are less inclined to receive booster shots, or are even less likely to vaccinate their offspring [13]. As a result, several countries, including some African countries, have not yet achieved herd immunity [103]. The World Health Organization also identified vaccine hesitancy as one of the most critical health threats to the successful implementation of any future COVID-19-like vaccination program [105].

Several studies employed the TPB to study behavioral intentions to vaccinate against COVID-19 [65,77,98,106]. From a theoretical perspective, this study was the first systematic review and meta-analysis of quantitative studies that used the TPB as the theoretical framework to examine its constructs contributing to the intention to vaccinate against COVID-19. Our findings suggest that the TPB provides a useful framework for explaining and predicting COVID-19 vaccination intention. Thus, public awareness and educational programs aimed at promoting vaccine acceptance should consider using TPB as a framework with the focus on attitude, subjective norms, perceived behavioral control, and self-efficacy. 

Our findings revealed that the COVID-19 vaccination intention rate was relatively high (73.19%). This finding corroborates the previous reviews of Wang et al. [47] and Terry et al. [53], who reported overall vaccine acceptance rates of 73.31% and 73.3%, respectively. Renzi et al. [52] reported a relatively smaller pooled prevalence of COVID-19 vaccination acceptance rate (66%). Alarcón-Braga et al. [54] found a very high vaccination acceptance (78.0%) among the general population in Latin America and the Caribbean. These findings indicate that overall vaccination intention rate remained stable and did not increase from 2020 to 2021. Vaccine acceptance was lower among parents for their children (59.15%). Thus, information campaigns targeted at parents should focus on communicating the safety and efficacy of COVID-19 vaccines.

Our findings demonstrate that attitude was the strongest and most frequently demonstrated TPB construct influencing vaccination intention, followed by subjective norms, and perceived behavioral control. This finding supports previous research [76,80,97]. However, our finding also contradicts other studies [72,95,96,99], in which, among the TPB constructs, perceived behavioral control was the strongest predictor of behavioral intention to vaccinate against COVID-19. However, a few studies reported that subjective norms had a smaller [83,92,96,107] or a larger [98,100,101] effect than other TPB constructs. Interestingly, attitude was the weakest predictor of vaccination intention in four studies [95,99,100,101]. Future research should further clarify these mixed findings. 

Possible explanations for these inconsistent findings are that the strength of associations between TPB constructs and behavioral intention to vaccinate against COVDI-19 may vary across different contexts. For example, our results show that the effects of TPB constructs on vaccination intention vary across geographic regions and study populations. While attitude had large effect sizes in Asia, Europe, and Oceania, especially among the adult general population, parents, and patients, subjective norms had large effect sizes in Asia and Oceania, especially among parents and patients. Perceived behavioral control was found to be a key factor influencing vaccination intention in Africa. Our results also confirm that the association between TPB constructs and vaccination intention varied according to study population. While attitude predicted intention among the general population, subjective norms were a stronger predictor of vaccine acceptance among patients and healthcare workers than the general adult population. Perceived behavioral control was an influential predictor of behavioral intention to vaccinate against COVID-19 among healthcare workers. These findings support a need to create messages tailored to specific target populations. More targeted communication strategies can be developed for the vaccine-hesitant populations.

Another explanation for the conflicting results may be presented in terms of data collection year. Our review revealed that subjective norms were a more dominant predictor of vaccine acceptance in 2020 than in 2021. On the contrary, attitude was a significant predictor of vaccine acceptance more frequently in 2021 than in 2020. Similarly, perceived behavioral control was found to influence vaccination intention more frequently in 2021 than in 2020. Therefore, to increase COVID-19 vaccination, people’s belief about the outcomes of vaccination and their perceptions of ability to control factors that hinder vaccination intention should be focused on. 

Over half of the studies that examined the association between perceived behavioral control and vaccination intention reported insignificant associations [69,73,91], threatening the TPB’s utility in predicting vaccination intention. A possible reason for the insignificant result could be the types of samples used by the studies. For example, perceived behavioral control had no effect among patients [62] and students [70,77]. Another possible explanation might be the geographical differences. Our study shows that the influence of perceived behavioral control was weaker in non-African countries. This warrants further investigation into the effect of perceived behavioral control on vaccination intention.

To sum up, findings from this investigation provide important insights for public health interventions on how to increase the coverage of vaccination, which is essential to reduce the load of DALYs (disability-adjusted life years) due to COVID-19, and to decrease the mortality rate. As a result of the mass vaccination drive, gradually, the world is overcoming the hazardous effect of the recent pandemic of COVID-19; but, the recurrence of similar and even worse pandemic cannot be denied. Hence, this systematic review and meta-analysis will be helpful to the agencies involved in vaccination, as well as prevention and control of pandemics. 

Vaccination intention is a complicated and multifaceted phenomenon, as well as a dynamic social process. This entails the existence of cognitive, psychological, sociodemographic, and cultural factors. Our results suggest that all TPB constructs are useful tools in promoting vaccination. However, several studies reported statistically insignificant effects of perceived behavior control and subjective norms on COVID-19 vaccination intention. This indicates that the predictive utility of the TPB may be different depending on various factors, including culture, country, target population, and study context. Our results also show that the impacts of TPB constructs on vaccination intention are determined by geographical differences, study population, and study year. Therefore, governments, policymakers, NGOs, and other stakeholders should consider these factors in developing interventions aimed at enhancing people’s positive attitudes toward vaccines, their perceptions of social pressure from their significant others to get vaccinated, and their perceived ability to get vaccines. Effective communication strategies may include encouragement from loved ones and trusted figures such as physicians and religious leaders, sharing personal stories, and peer pressure. Minority, lower-income, and less-educated individuals are disproportionately more susceptible to COVID-19 [107,108]. They also have lower acceptance, which requires special attention, addressing the effect of their chronic distrust of health authorities in order to confront the vicious cycle of skepticism. In policy planning to combat the pandemic, caution should be taken in interpreting and using the results, since intention or survey responses may not directly predict future behavior [109]. Moreover, opinions may change, especially amid the raging pandemic. Reported clinical trials incidents or outcomes and subsequent introductions of vaccines or new treatments would further change people’s minds about getting vaccinated. Hence, policymakers must review their strategy in definite intervals. Since several factors affect individuals’ decision to accept a COVID-19 vaccine, a holistic educational approach to improve confidence in the COVID-19 vaccine should be implemented. Moreover, policymakers should develop and implement targeted education for people with a low level of knowledge that are designed to increase their self-efficacy (i.e., confidence in their ability to receive the vaccines or to overcome vaccination barriers). This study reveals that there exist noticeable psychological, demographic, and geographical disparities in vaccine acceptance. Hence, a country- and population-specific strategy is required for successful mass vaccination drive and to attain herd immunity.

This systematic review and meta-analysis identified several important areas for future research. First, nearly one-fifth of the studies (18.6%) included in this study focused on only one or two of the TPB constructs. Moreover, only three studies [63,73,78] examined the role of moderators and mediators in the relationships between TPB constructs and COVID-19 vaccination intention. Thus, future studies should consider extending the TPB model by incorporating various factors such as mediators, moderators, covariates, and confounders. Second, the studies included in this systematic review and meta-analysis were conducted only in twenty countries, mostly in developed or emerging nations, which focused on the general adult population, patients, students, healthcare workers, parents, and factory workers. Hence, future studies should examine the applicability of the TPB model in predicting vaccine acceptance using diverse samples from understudied countries. Third, our systematic review and meta-analysis also showed that only two studies investigated parental acceptance of COVID-19 vaccination for their children. Hence, more research is needed to understand the applicability of the TPB model for understanding parental vaccine acceptance. Fourth, a vast majority of the studies included in our study were cross-sectional in design. In addition, only two studies [8,80] investigated the associations between TPB constructs and COVID-19 vaccine acceptance for booster shots. Therefore, more longitudinal studies are needed to gain a better understanding of how TPB predicts vaccination intention over time. Finally, the subgroup meta-analytic results of the current study need to be interpreted with caution as the percentage of variability attributed to heterogeneity for most subgroups remained high, indicating that the samples in the included studies were heterogeneous. Therefore, future studies should consider performing subgroup analyses or meta-regression analyses by incorporating other moderating variables. 

## 6. Conclusions

This systematic and meta-analytic review represents an initial attempt to analyze the literature using the TPB as a theoretical model to examine the influence of TPB constructs on vaccination intention against COVID-19. Attitude was the strongest predictor of vaccination intention, followed by subjective norms and perceived behavioral control. However, the effects of these TPB constructs on behavioral intention to vaccinate against COVID-19 were moderated by geographic region and study population. These findings provide important insights for developing health education messages to promote acceptance of vaccination against COVID-19.

## Figures and Tables

**Table 1:** Search Strategy.

Search	Search Terms (Boolean Operators)
#1	“theory of planned behav*” AND “vaccination intent*” OR vaccine accept*” AND “COVID-19”
#2	“theory of planned behav*” AND “vaccination intent*” OR vaccine accept*” AND “coronavirus”
#3	“theory of planned behav*” AND “vaccination intent*” OR vaccine accept*”AND “SARS-CoV-2”
#4	“theory of planned behav*” AND “vaccin* intent*” OR “vaccin* accept*” AND “COVID-19” OR “coronavirus” OR “SARS-CoV-2”

**Table 3:** The Meta-analysis Results of Subgroup Analyses (Attitude–Intention).

Group	# of Studies	Effect Size (95% CI)	Z-Value	*p*-Value	Q-Value	*p*-Value	I^2^
Continent							
Africa	2	0.33 [0.28, 0,37]	13.34	0.00	12.92	0.00	92.26
Asia	18	0.65 [0.64, 0.66]	148.90	0.00	6362.99	0.00	99.73
Europe	7	0.63 [0.62, 0.63]	81.05	0.00	1903.42	0.00	99.68
North America	9	0.34 [0.32, 0.35]	33.65	0.00	1643.74	0.00	99.51
Oceania	2	0.70 [0.67, 0.72]	30.86	0.00	9.57	0.00	89.56
Total within					9932.65	0.00	
Total between					1632.95	0.00	
Population							
Adult general	27	0.58 [0.57, 0.58]	129.28	0.00	5941.28	0.00	99.56
Factory worker	2	0.10 [0.07, 0.13]	6.28	0.00	9.44	0.00	89.41
Healthcare worker	2	0.30 [0.26, 0.36]	11.18	0.00	6.31	0.01	84.15
Parent	1	0.84 [0.83, 0.85]	128.88	0.00	0.00	1.00	0.00
Patient	3	0.53 [0.47, 0.57]	16.54	0.00	290.81	0.00	99.31
Student	3	0.39 [0.36, 0.41]	27.72	0.00	156.97	0.00	98.73
Total within					6404.81	0.00	
Total between					5160.79	0.00	

**Table 4:** The Meta-analysis Results of Subgroup Analyses (Subjective Norms–Intention).

Group	# of Studies	Effect Size (95% CI)	Z-Value	*p*-Value	Q-Value	*p*-Value	I^2^
Continent							
Africa	2	0.25 [0.20, 0.29]	10.00	0.00	141.79	0.00	99.29
Asia	19	0.52 [0.51, 0.53]	110.42	0.00	3731.04	0.00	99.52
Europe	8	0.17 [0.16, 0.19]	19.41	0.00	1318.08	0.00	99.47
North America	7	0.27 [0.25, 0.29]	24.25	0.00	1116.75	0.00	99.46
Oceania	2	0.62 [0.59, 0.66]	26.35	0.00	12.51	0.00	92.01
Total within					6320.19	0.00	
Total between					1948.53	0.00	
Population							
Adult general	26	0.41 [0.40, 0.42]	84.38	0.00	5710.76	0.00	99.56
Factory worker	2	0.24 [0.21, 0.26]	15.41	0.00	85.40	0.00	98.83
Healthcare worker	2	0.35 [0.30, 0.40]	12.93	0.00	94.64	0.00	98.94
Parent	1	0.63 [0.62, 0.64]	78.25	0.00	0.00	1.00	0.00
Patient	4	0.59 [0.56, 0.63]	23.37	0.00	183.86	0.00	98.37
Student	3	−0.01 [−0.04, 0.02]	−0.78	0.43	3.75	0.15	46.61
Total within					6078.42	0.00	
Total between					2190.30	0.00	

**Table 5:** The Meta-analysis Results of Subgroup Analyses (PBC–Intention).

Group	# of Studies	Effect Size (95% CI)	Z-Value	*p*-Value	Q-Value	*p*-Value	I^2^
Continent							
Africa	2	0.46 [0.42, 0.50]	19.67	0.00	88.76	0.00	98.87
Asia	15	0.38 [0.37, 0.39]	74.76	0.00	1089.85	0.00	98.72
Europe	7	0.09 [0.08, 0.11]	10.06	0.00	663.56	0.00	99.10
North America	6	0.04 [0.01, 0.06]	3.09	0.00	12.08	0.03	58.60
Total within					1854.25	0.00	
Total between					1370.10	0.00	
Population							
Adult general	20	0.27 [0.26, 0.28]	51.13	0.00	2838.61	0.00	99.33
Factory worker	2	0.13 [0.10, 0.16]	8.12	0.00	6.78	0.01	85.25
Healthcare worker	2	0.37 [0.32, 0.42]	13.68	0.00	37.62	0.00	97.34
Parent	1	0.36 [0.34, 0.38]	39.78	0.00	0.00	1.00	0.00
Patient	3	0.43 [0.37, 0.48]	12.83	0.00	112.90	0.00	98.23
Student	2	0.28 [0.25, 0.31]	18.21	0.00	0.00	0.96	0.00
Total within					2995.92	0.00	
Total between					228.44	0.00	

## Data Availability

Data generated in this study are available by contacting the first author, Yam B. Limbu, if requested reasonably.
